# Rapid removal of Pb^2+^ from aqueous solution by phosphate-modified baker's yeast

**DOI:** 10.1039/c7ra13545a

**Published:** 2018-02-20

**Authors:** Shuli Liu, Zhengyang Duan, Changhua He, Xiaojun Xu, Tianguo Li, Yuhuan Li, Xuan Li, Yao Wang, Longqian Xu

**Affiliations:** Faculty of Environmental Science and Engineering, Kunming University of Science and Technology Kunming Yunnan 650500 China xuxiaojun88@sina.com +86-13577132038; College of Resources and Environment, Yunnan Agricultural University Kunming Yunnan 650201 China

## Abstract

Phosphate-modified baker's yeast (PMBY) was prepared, and used as a novel bio-sorbent for the adsorption of Pb^2+^ from aqueous solution. The influencing factors, absorption isotherms, kinetics, and mechanism were investigated. The scanning electron microscopy (SEM), Fourier-transform infrared spectroscopy (FTIR) characterization and elemental analysis of PMBY showed that phosphate groups were successfully grafted onto the surface of yeast. The kinetic studies suggested that the adsorption process followed a pseudo-second-order chemisorption. The adsorption process of Pb^2+^ using PMBY was spontaneous and endothermic. Furthermore, the adsorption of Pb^2+^ on PMBY can rapidly achieve adsorption equilibrium (in just 3 min), and the maximum adsorption capacity of Pb^2+^ on PMBY was found to be 92 mg g^−1^ at 30 °C, which was about 3 times that of the pristine baker's yeast. The suggested mechanism for Pb^2+^ adsorption on PMBY was based upon ion-exchange, electrostatic interaction and chelation between the phosphate groups and Pb^2+^. However, compared with the pristine baker's yeast, the higher capacity and rapid adsorption of PMBY for Pb^2+^ was mainly due to the chelation and electrostatic interactions between the phosphate groups and Pb^2+^. In addition, the regeneration experiments indicated that the PMBY was easily recovered through desorption in 0.01 M HCl, and that PMBY still exhibited 90.77% of the original adsorption capacity for Pb^2+^ after five regeneration cycles. These results showed the excellent regeneration capability of PMBY for Pb^2+^ adsorption. PMBY has shown significant potential for the removal of heavy metals from aqueous solution due to its rapid adsorption, high-capacity and facile preparation.

## Abstract 2

Phosphate-modified baker's yeast (PMBY) was used as a novel bio-sorbent for the adsorption of Pb^2+^ from aqueous solution.

## Graphical abstract

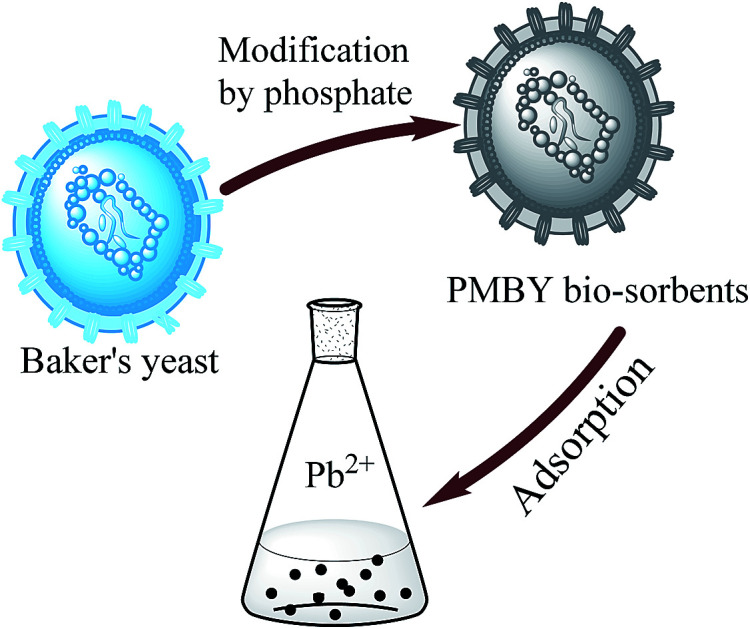

## 1. Introduction

Lead is widely used in various fields, such as lead-acid batteries, construction materials, printing, pigments, fossil fuels, photographic materials, and manufacturing of explosives.^1,2^ However, excessive discharge of lead to the environment can damage the ecosystem due to its highly poisonous nature towards living organisms. Lead possesses non-biodegradable features, and easy accumulation in the human body through the food chain, particularly when it is discharged into aquatic environments.^3^ It is well known that lead exposure could cause severe health problems, such as physiological and neurological disorders, especially in children even at low lead concentrations.^4–6^ Lead is classified as a priority pollutant by the US Environmental Protection Agency (EPA). In addition, the permissible levels of Pb^2+^ in drinking and wastewater are 0.05 mg L^−1^ and 0.005 mg L^−1^, respectively.^7^

Considering the hazards associated with lead, a method involving highly efficient separation and recovery of lead from contaminated water is of great significance not only for the full utilization of lead resources, but also to protect the human health and ecological environment. Many methods have been used to treat wastewater containing lead, including chemical precipitation, electrochemical treatment, reduction, ion-exchange, solvent extraction, adsorption and flotation.^8–10^ There are some disadvantages associated with most of these methods, which restrict their application. These disadvantages include low efficiency, high energy consumption, large quantity of toxic and expensive materials used, and production of large amounts of sludge, which needs secondary treatment in some methods.^8,11^ Nevertheless, bio-adsorption has attracted considerable attention due to its environment-friendly nature and low cost. Additionally, bio-adsorption can effectively remove soluble and insoluble pollutants without generating hazardous by-products.^12^

Various microorganisms, such as bacteria, fungi and algae are a kind of bio-sorption materials, which can adsorb heavy metal ions.^13–15^ For bio-adsorption technology, the selection of appropriate biomaterial for the removal of hazardous heavy metals from aqueous solutions is a key process step.^8^ The source, safety, cost and adsorption capacity should be considered for the selection of any suitable biomaterial. Among the aforementioned biomaterials, yeast cells are frequently-used fungi, which often serve as suitable sources of bio-sorbent materials due to their easy cultivation, and have features such as inexpensive large-scale growth media, wide availability and safety.^16,17^ Previous researchers have demonstrated that the surface of yeast cells contains abundant amounts of functional groups, which can adsorb heavy metals, such as hydroxyl, carbonyl, and amide groups. However, the sorption capacities of yeast cells are still unsatisfactory due to limited surface functional groups.^18^ Therefore, it is necessary to improve the adsorption performance of yeast cells, especially with regards to the adsorption of lead. A number of modified strategies, such as the formation of nano-MnO_2_/nano-ZnO and hydroxyapatite on the yeast surface,^19–21^ modification of EDTAD/ethylenediamine/polymer,^22–24^ and pretreatment using ethanol/caustic have been proposed to improve the adsorption capacity of yeast.^25^ Surface modifications of yeast with organic and inorganic materials provide a hybrid material having higher efficiency and capacity for the removal of heavy metals by either introducing or exposing more surface functional groups on the surface of raw materials.^26^ Although, the aforementioned modifications of yeast improved the adsorption capacity for heavy metals, their relatively complicated synthesis and difficult procurement of preparation materials led to high costs. Therefore, synthesizing new bio-sorbents was more competitive and practical among various bio-sorbents, which have the capacity to sequester the heavy metal ions from aquatic environment. To achieve this, it is necessary to fabricate low-cost, reliable, rapid adsorption, durable and efficient materials. Among these properties, the rapid adsorption of bio-sorbents is one of the most serious problems hindering the commercial application of bio-sorbents. Many bio-sorbents need a long time to reach adsorption equilibrium, which would result in significant waste of energy and hence, reduce the treatment efficiency. Therefore, considering the adsorption rate while synthesizing a novel bio-sorbent is highly important for the overall efficiency of the adsorption process.

Phosphate is an inorganic material that is non-toxic and inexpensive. Phosphate groups are known to have excellent chelating properties for metal ions. Thus, many phosphorylated materials were applied to removal metal ions. For example, phosphorylated cellulose microspheres,^27^ phosphorylated chitosan,^28^ and phosphorylated starch have been used as adsorbents for metal ions removal.^29^ To the best of our knowledge, phosphate-modified baker's yeast has not been investigated in detail for the removal of lead from aqueous solutions. By forming hydroxyapatite on the surface of yeast, the functional groups of pristine yeast do not participate in the synthesis reaction. In other words, it is worth studying whether the phosphate-modified baker's yeast, which *via* the interaction between the phosphate and surface functional groups of baker's yeast, is a feasible and effective means to obtain an efficient and cheap bio-sorbent for Pb^2+^ or not.

Herein, a phosphate-modified baker's yeast (PMBY) was prepared using a simple pathway that involved phosphate treatment of baker's yeast and dry-heating. Then, the adsorption characteristics, kinetics, and isothermal behavior of PMBY for Pb^2+^ adsorption from aqueous solution were explored. Subsequently, a comparative analysis along with the scanning electron microscopy (SEM), Fourier-transform infrared spectroscopy (FTIR), and X-ray photoelectron spectroscopy (XPS) analyses were conducted to further explore the adsorption performance and mechanism of PMBY.

## 2. Materials and methods

### 2.1 Materials

The commercially fresh baker's yeast was supplied by Angel Yeast Co., Ltd., China, and was repeatedly washed with deionized water to remove adhering dirt and soluble impurities. The resulting yeast was dried at 80 °C for 24 h, and then, crushed and sieved to a particle size of less than 100 mesh. The resulting purified yeast was named as the pristine baker's yeast.

Various chemicals and reagents, including sodium dihydrogen phosphate (NaH_2_PO_4_·2H_2_O), sodium hydrogen phosphate (Na_2_HPO_4_·12H_2_O), sodium hydroxide (NaOH), nitric acid (HNO_3_), lead nitrate (Pb(NO_3_)_2_), and ammonium molybdate ((NH_4_)_6_Mo_7_O_24_·4H_2_O) were purchased from Aladdin-Biochemical Technology Co., Ltd., China. All these chemicals were of analytical reagent grade, and used without further purification. Lead nitrate was employed as the Pb^2+^ source. The stock standard solution of Pb(NO_3_)_2_ was obtained from the National Analysis Center for Iron and Steel (Beijing, China). The working solutions were obtained by diluting the stock solution. Furthermore, 1 M NaOH and 1 M HNO_3_ were used to adjust the pH values. All solutions were prepared using deionized water.

### 2.2 Preparation of phosphate-modified baker's yeast

**Fig. 1:**
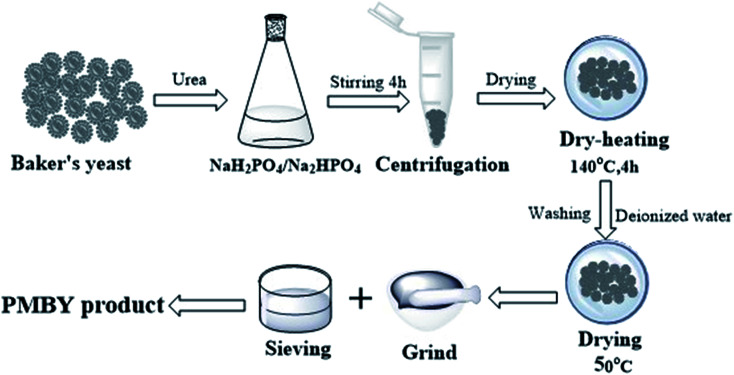
Synthesis of PMBY.

7.5 g phosphates including 3.49 g NaH_2_PO_4_ and 4.01 g Na_2_HPO_4_ (the mass ratio of NaH_2_PO_4_·2H_2_O : Na_2_HPO_4_·12H_2_O was 0.87 : 1 (ref. 29), respectively) was dissolved in 100 mL deionized water. Then 5.0 g of baker's yeast and 0.01 g urea were added in the above solution. The pH of the mixture was adjusted to 6 using a pH meter (PHSJ-4F, China), and the mixture was stirred continuously (200 rpm; 4 h) at room temperature. It was then centrifuged at 4 °C, with 1000 rpm for 10 min through high speed freezing centrifuge (GL-21M, China). The solid was dried at 50 °C under 0.7 MPa pressure in a vacuum drying oven (DZF-6050, China) until the moisture content was less than 15 wt%. The dried product was incubated at 140 °C for 4 h in a vacuum drying oven, after which, the product was washed using deionized water. Then, it was centrifuged until there was no change in color of the liquid that was obtained after the centrifugation. (NH_4_)_6_Mo_7_O_24_·4H_2_O was added and the mixture was heated at around 60–70 °C in a thermostatic water bath (HJ-M6, China). Finally, the product was ground in an agate mortar (YXY-A01, China) and sieved to a particle size of less than 100 mesh using a standard sieve. The product was dried in a vacuum drying oven at 50 °C under 0.7 MPa for 10 h before further use. The detailed synthesis process is shown in Fig. 1.

### 2.3 Characterization

The X-ray powder diffraction (XRD) patterns were recorded on an X'Pert^3^ Powder diffractometer (PANalytical B. V., The Netherlands) using Cu Kα radiation (*λ* = 1.54 Å, 40 kV, 40 mA) over 2*θ* range of 5–90° with a resolution of 0.026°. The scanning speed was 8.0° min^−1^ and the measurements were conducted at ambient temperature.

The morphology and the elemental composition of the samples were studied using tungsten filament scanning electron microscopy (SEM) and energy dispersive spectrometer (EDS) (JSM-7500F, Japan), operated at 20 kV acceleration voltage. Fourier-transform infrared spectra (FTIR) was observed using a PerkinElmer spectrometer (L1600400 spectrum Two DTGS, USA), which used potassium bromide (KBr) pellets. The mass ratio of potassium bromide to sample was 700 : 1, respectively. The FTIR analysis was obtained within the range of 400–4000 cm^−1^.^30,31^ The elemental analyses (C, H, O and N) were performed on an elemental analyzer (Elementar Vario Micro Cube, Germany). Moreover, the phosphorus content was assayed following the Chinese National Standard (GB 5009.268-2016), and was analyzed using a UV/Vis spectrophotometer (UV-VIS752, China) at 660 nm wavelength. X-ray photoelectron spectroscopy (XPS) was used to analyze the surface elemental composition of the samples. The measurements were carried out using Kratos Axis Ultra DLD (SHIMADZU, Japan) at room temperature. The ejected photoelectrons used a monochromatic beam of Al Kα X-rays (*hν* = 1486.6 eV) and the resulting binding energy peaks were referenced to C1s peak occurring at 284.8 eV. N_2_ adsorption–desorption isotherms were measured using a surface area analyzer (JW-BK132F, China). The specific surface area and pore size distribution of the samples were determined using Brunauer–Emmett–Teller (BET) method and Barrett–Joyner–Halenda (BJH) model.

### 2.4 Batch adsorption studies

Adsorption experiments were conducted under various conditions of pH, PMBY dosage levels, initial concentrations of lead ions, contact times and temperatures. For the sorption process, 100 mL of simulated Pb^2+^ solution with different initial concentrations (ranging between 25–250 mg L^−1^) were added to a series of 250 mL conical flasks. After a certain amount of PMBY was added to the Pb^2+^ solutions and the pH adjusted to a specified value, the mixture was agitated using a rotary shaker (speed of 150 rpm) for a specified time (*t*, min) at a specified temperature (*T*, °C). After reaching equilibrium, the mixtures were filtered through 0.45 μm filter membrane, and the filtrate was used to determine the Pb^2+^ concentration using atomic adsorption spectrophotometer (AAS, Hitachi, Z-5000, Japan). In this work, all adsorption experiments were performed in triplicates, and the average values were used to report the results.

The removal efficiency and the adsorption capacity of PMBY for Pb^2+^ were represented by *R* (%) and *q*_e_ (mg g^−1^), respectively, and were calculated using eqn (1) and (2), respectively.1
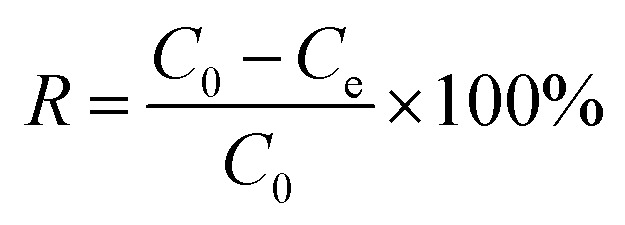
2
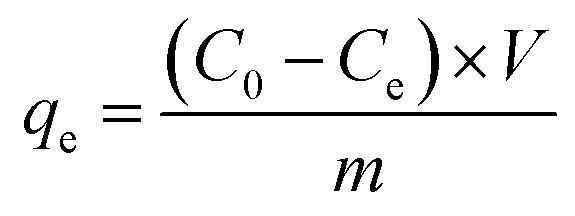
where *C*_0_ and *C*_e_ are the initial and equilibrium concentrations of Pb^2+^ in the solution (mg L^−1^), respectively, *V* is the volume of the testing solution (L), and *m* is the amount of bio-sorbent PMBY (g).

### 2.5 Regeneration of PMBY

To evaluate the regeneration of as-obtained PMBY, the cycle number-dependent adsorption capacities were analyzed for 100 mg L^−1^ Pb^2+^. The saturated PMBY loaded with Pb^2+^ was dispersed in various eluents (0.01 HCl, HNO_3_ and H_2_SO_4_). Afterwards, the solid materials were collected by centrifuging at 10 000 rpm for 20 min, washed thoroughly with deionized water, and then, reused in the next run of adsorption experiments.

## 3. Results and discussion

### 3.1 Characterization of PMBY

**Fig. 2:**
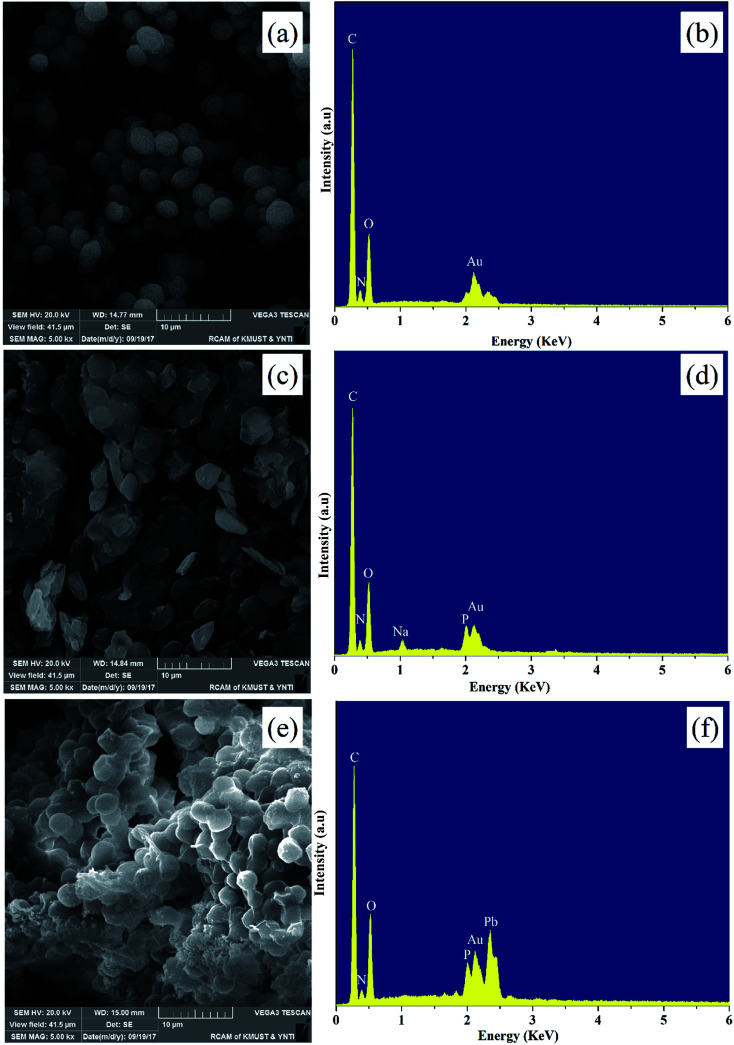
SEM images and EDS spectra of pristine baker's yeast (a and b), PMBY (c and d) and PMBY-Pb (e and f).

SEM analysis is a useful tool for characterizing the surface morphology of biosorbents. The PMBY exhibited clear differences in morphology relative to the pristine baker's yeast, as can be seen from Fig. 2a and c. The pristine baker's yeast was approximately spherical or ellipsoidal with the diameter of around 3–4 μm, while the surface was smooth and regular. After the phosphate modification, the PMBY displayed irregular shape and a large volume of pores was formed due to the aggregation of cells, which could prove beneficial to the adsorption of lead ions from aqueous solution. In addition, the corresponding Energy Dispersive Spectrometer (EDS) patterns (Fig. 2b and d) were used to characterize the basic elements on the surface of pristine baker's yeast and PMBY. As can be seen from Fig. 2b and d, the new peaks of P and Na appeared on PMBY except for the peaks of C, N and O, which were also present in the original yeast. The present form of phosphorus and the introducing mechanism were further studied using the FTIR spectra. The existence of gold elements was attributed to the samples, which were gold-coated with a thin layer of gold before the SEM analysis.

**Fig. 3:**
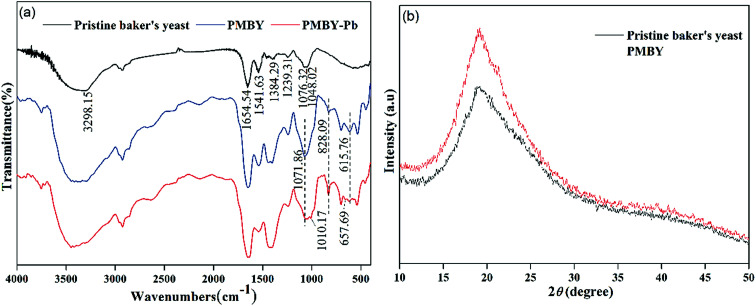
(a) FTIR spectra of pristine baker's yeast, PMBY and PMBY-Pb. (b) XRD patterns of pristine baker's yeast and PMBY.

Fig. 3a shows the FTIR spectra of baker's yeast and PMBY. The FTIR spectra of pristine baker's yeast consisted of typical peaks of hydroxyl (3298.15 cm^−1^),^20^ carboxyl (1384.29 cm^−1^),^24^ amine-I (1654.54 cm^−1^), amide-II (1541.63 cm^−1^), amide-III (1239.31 cm^−1^), and phosphate groups (1048.02 cm^−1^).^32–34^ Compared with the pristine baker's yeast (shown in Fig. 3a), some changes were observed in the FTIR spectra of PMBY. The peaks at 828.09 and 615.76 cm^−1^ represented the P–O–C aliphatic bonds and symmetric stretching vibration of PO_4_, respectively.^27,35^ The new peaks at 828.09 and 615.76 cm^−1^ coincided with the phosphate group,^36^ and the two peaks at 1048.02 and 1076.32 cm^−1^ presented in the pristine baker's yeast merged into one peak at 1071.86 cm^−1^, which was assigned to P–O vibration, while its intensity increased remarkably.^37^ These changes indicated that the phosphate groups were successfully grafted on the surface of yeast. Besides, the peak height and peak band of hydroxyl, carboxyl and amine groups of pristine baker's yeast changed after the phosphate modification, which indicated that these groups had participated in the reaction.

The phosphate groups, which were linked to the yeast, may have appeared due to either the substitution reaction or the ligand exchange process between the O–H group of hydroxyl groups and carboxylic acids, and phosphate. This can be represented using reaction eqn (3)–(6).

3

<svg xmlns="http://www.w3.org/2000/svg" version="1.0" width="23.636364pt" height="16.000000pt" viewBox="0 0 23.636364 16.000000" preserveAspectRatio="xMidYMid meet"><metadata>
Created by potrace 1.16, written by Peter Selinger 2001-2019
</metadata><g transform="translate(1.000000,15.000000) scale(0.015909,-0.015909)" fill="currentColor" stroke="none"><path d="M80 600 l0 -40 600 0 600 0 0 40 0 40 -600 0 -600 0 0 -40z M80 440 l0 -40 600 0 600 0 0 40 0 40 -600 0 -600 0 0 -40z M80 280 l0 -40 600 0 600 0 0 40 0 40 -600 0 -600 0 0 -40z"/></g></svg>

R–OH + HPO_4_^2−^ → R–O–PO_3_^2−^ + H_2_O

4 R–OH + H_2_PO_4_^−^ → R–O–HPO_3_^−^ + H_2_O

5 R–COOH + HPO_4_^2−^ → R–CO–O–PO_3_^2−^ + H_2_O

6 R–COOH + H_2_PO_4_^−^ → R–CO–O–HPO_3_^−^ + H_2_O

where R represents the surface.

Additionally, the amine groups and phosphate groups could react through electrostatic attraction and hydrogen bonding.

The XRD patterns of pristine baker's yeast and PMBY composites are shown in Fig. 3b. Pristine baker's yeast presented a broad strong peak at about 2*θ* of 20°. In contrast to the pristine baker's yeast, the PMBY composites not only showed stronger diffraction pattern at about 2*θ* of 20°, but also exhibited few well-defined peaks involving crystal phosphate. These results suggested that the phosphate in PMBY composites may be in a non-stoichiometric and amorphous phase.^20^ The results were assigned to the content of phosphate in PMBY, which did not reach XRD's detection limit (5 wt%), whereas the crystallization of these was poor and not within the detectable range.^38^

The elemental analysis of the samples show that PMBY contains 45.02% C, 34.470% O, 8.160% H, 8.41% N and 0.53% P, respectively. The contents of C and H of PMBY decreases by comparing with pristine baker's yeast (C: 39.01%, O: 41.135%, H: 7.363%, N: 8.36%, P: 4.06%), while the contents of O and P increase significantly after reacting with phosphate. The results confirm that PMBY had been successfully synthesized.

### 3.2 Adsorption behavior of PMBY for Pb^2+^

#### 3.2.1 Effect of pH

**Fig. 4:**
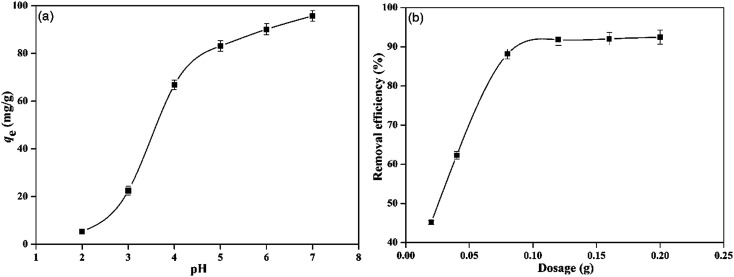
Effect of pH (a) and PMBY dosage (b) on the adsorption of Pb^2+^.

Solution pH is one of the most important environmental factors affecting the sorption of metallic ions. To observe the influence of pH on Pb^2+^ adsorption, adsorption experiments under various pH values were conducted (*C*_0_ = 100 mg L^−1^, PMBY dosage = 0.08 g, *V* = 100 mL, *t* = 30 min, *T* = 30 °C and pH = 2.0–7.0), and the results are shown in Fig. 4a. The adsorption of Pb^2+^ increased rapidly from 5.39 to 83.14 mg g^−1^ with the increase in pH from 2.0 to 5.0, respectively. The pH-dependence indicated that the bio-sorption capacities of Pb^2+^ on PMBY were affected by the surface complexation. When the solution pH values were within the range of 2.0–3.0, relatively low adsorption capacity was observed, which could be attributed to the protonation of active sites and the competition between the H^+^ and Pb^2+^ for binding sites.^6^ As the pH increased from 3.0 to 5.0, the H^+^ ions left the surface of bio-sorbent PMBY, and decreased the protonation of functional groups to improve the adsorption capacity. In addition, the optimum uptake was observed at the pH value of 5.0 due to the presence of ligands (such as, carboxyl, amide and phosphate groups) on the surface of sorbent, which have p*K*_a_ values within the range of 3–5 (ref. 39). However, at higher pH values (pH > 6.0), Pb^2+^ will precipitate out of the solution, and therefore, it is difficult to judge whether the adsorption or the precipitation has taken place. Hence, the optimum initial pH value of 5.0 was used in all further experiments.

#### 3.2.2 Effect of dosage of PMBY bio-sorbent

The removal of Pb^2+^ using PMBY at various dosages was investigated (*C*_0_ = 50 mg L^−1^, pH = 5.0, *T* = 30 °C, PMBY dosage = 0.02–0.20 g, *V* = 100 mL and *t* = 30 min), and the results are shown in Fig. 4b. It was observed that the adsorption efficiency sharply increased from 45.15% to 88.16% as the PMBY dosage increased from 0.02 to 0.08 g, respectively, which was due to the reason that the surface area and binding sites of PMBY (available to Pb^2+^) increased accordingly as the sorbent's dosage increased. When the PMBY dosage increased from 0.08 to 0.2 g, the adsorption efficiency for Pb^2+^ only increased by 4.8%. Due to a small increase, the PMBY dosage of 0.08 g was chosen to conduct further experiments.

#### 3.2.3 Adsorption kinetics

**Fig. 5:**
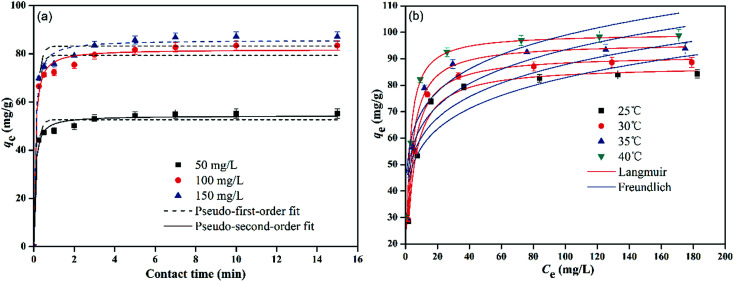
(a) Kinetic behavior and (b) adsorption equilibrium of PMBY adsorption for Pb^2+^.

The effect of contact time on the adsorption capacity of PMBY for Pb^2+^ was investigated (*C*_0_ = 50, 100, 150 mg L^−1^, pH = 5.0, *T* = 30 °C, PMBY dosage = 0.08 g, *V* = 100 mL), and the results are presented in Fig. 5a. The results show that the rate of adsorption of PMBY for Pb^2+^ was high, and required only around 3 min to reach equilibrium. The rapid interaction of sorbent with the targeted metallic ions is desirable and beneficial for practical adsorption applications. The rapid rate of uptake indicated that the surface of PMBY had plenty of vacant active sites for the sorption of lead ions. After the first 3 minutes, the adsorption became difficult due to repulsive forces between the adsorbed lead ions on PMBY surface and the lead ions in the bulk solution.^40^ Considering the practical operation, the optimal time was selected as 15 min for further analysis in this work. The pseudo-first-order (eqn (7)) and pseudo-second-order (eqn (8)) kinetic models were introduced to determine the adsorption kinetics of Pb^2+^.^39^

7 *q*_*t*_ = *q*_e_(1 − e^−*k*_1_*t*^)

8
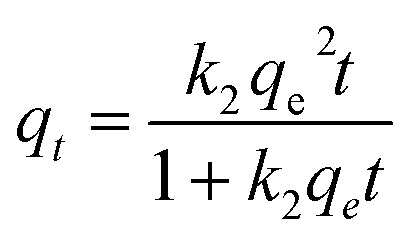
where *q*_*t*_ is the amount adsorbed at time *t* (min) in mg g^−1^, and *k*_1_ (min^−1^) and *k*_2_ (g mg^−1^ min^−1^) represent the adsorption rate constants for pseudo-first-order and pseudo-second-order, respectively. The fitting results are presented in Fig. 5a and Table 1.

**Table 1:** Kinetic parameters for the adsorption of PMBY for Pb^2+^

*C*_0_ (mg L^−1^)	Experimental (mg g^−1^)	Pseudo-first-order			Pseudo-second-order		
		*K*_1_	*q*_e_	*r* ^2^	*K*_2_	*q*_e_	*r* ^2^
50	55.20	6.71	52.67	0.9724	0.27	54.31	0.9907
100	83.41	6.67	79.34	0.9713	0.18	81.84	0.9902
150	87.11	6.71	83.14	0.9725	0.17	85.72	0.9908

The calculated correlation coefficient values (*r*^2^) for pseudo-first-order and pseudo-second-order kinetics were found to be higher than 0.97, which show that both kinetic models can be used to predict the adsorption behavior of Pb^2+^ using PMBY for the entire contact time (Table 1). The predicted *q*_e_ values at different Pb^2+^ concentrations using pseudo-second-order model were in a better agreement with the experimental values than the pseudo-first-order, which indicated that the adsorption process could be explained using pseudo-second-order model, while the adsorption rate was controlled by chemisorption.^41–43^ In addition, the pseudo-second-order rate constant (*k*_2_) decreased as the Pb^2+^ concentration increased from 50 to 150 mg L^−1^, suggesting that it took longer to achieve the adsorption equilibrium at higher Pb^2+^ concentrations, which may have been due to the limited number of available active sites on PMBY.

It is interesting to observe that, PMBY not only efficiently removed Pb^2+^ from the aqueous solution, but it also resulted in a better and faster removal rate than some other bio-sorbents. In order to display the advantage of PMBY, the maximum adsorption capacity of PMBY at 30 °C and the equilibrium time were compared with various yeast-based bio-sorbents used for Pb^2+^ adsorption (Table 2). The results indicated that the PMBY had relatively better adsorption capacity than the most of reported yeast-based bio-sorbents. Although the adsorption capacity of PMBY is lower than some bio-sorbents reported in literature (Table 2), the adsorption equilibrium time was very short compared with other reports. The rapid adsorption of PMBY makes it competitive to various other bio-sorbents.

**Table 2:** Comparison of adsorption capacities and equilibrium time of various yeast-based bio-sorbents for Pb^2+^

Bio-sorbents	Biosorption capacity (mg g^−1^)	Equilibrium time (min)	Reference
Bakers' yeast	28.45	30	This work
PMBY	91.53	3	This work
Nano-ZnO/yeast composites	31.72	30	19
HAP/yeast biomass composites	48.93	60	21
EMB	99.26	30	22
Ethanol treated baker's yeast	17.49	120	44
Polymer modified baker's yeast	203.06	20	45
Cystine-modified yeast	45.87	20	46
EYMC	127.37	60	23
Waste beer yeast	5.72	60	47

#### 3.2.4 Isothermal study

Fig. 5b shows the sorption isotherms for Pb^2+^ adsorbed on PMBY under the conditions of: pH = 5.0; PMBY dosage = 0.08 g; *V* = 100 mL, *t* = 15 min; *T* = 25 °C, 30 °C, 35 °C and 40 °C, and *C*_0_ ranging between 25–250 mg L^−1^. The results indicated that the sorption capacity of PMBY increased both with temperature and initial Pb^2+^ concentration. The *q*_e_ increased significantly at low Pb^2+^ concentrations, which indicated that the initial Pb^2+^ concentration played a critical role, which could produce a key driving force among lead ions to reduce the mass transfer resistance of lead between the liquid and solid phases, and hence, can enhance the effective collision probability between the lead ions and PMBY. The equilibrium adsorption capacity remained nearly constant even when the initial Pb^2+^ concentrations went past a certain value (100 mg L^−1^; in this work), which could be explained by the saturation of active sites on PMBY surface. These results suggest that the available active sites on PMBY were the limiting factor for the adsorption of lead ions. Meanwhile, the adsorption capacity of PMBY for Pb^2+^ increased from 84.26 to 98.77 mg g^−1^ with the increase in temperature from 25 to 40 °C, which indicated that the adsorption process was endothermic in nature.

To describe the sorption characteristics of PMBY more adequately, the equilibrium data from Fig. 5b was modeled using Langmuir and Freundlich isotherm models.^48^

The Langmuir isotherm model assumes homogeneous adsorption during the adsorption process. The Langmuir isotherm can be expressed using eqn (9).9
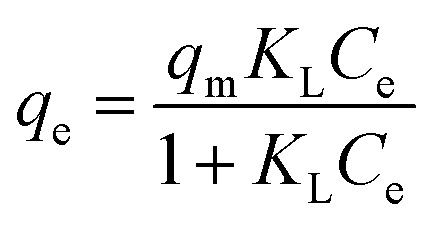
where *q*_m_ is the maximum amount of Pb^2+^ adsorbed by PMBY (mg g^−1^) and *K*_L_ is the Langmuir constant, which is related to the sorption energy (L mg^−1^).

The Freundlich isotherm model assumes a heterogeneous adsorption, and infers that the heavy metal ions, which have been bided on the surface sites, may affect the adjacent sites. The Freundlich isotherm is represented by eqn (10).

10 *q*_e_ = *K*_F_*C*_e_^1/*n*^

where *K*_F_ is the Freundlich constant related to the strength of interactions between Pb^2+^ and PMBY [(mg g^−1^) (L mg^−1^)^1/*n*^], and 1/*n* is the empirical parameter related to the adsorption intensity, which varies according to the heterogeneity of the sorbent.

Fig. 5b and Table 3 display the fitting results for Langmuir and Freundlich models, and show that the Langmuir isotherm could fit the equilibrium data better than the Freundlich isotherm. Firstly, the Langmuir isotherm resulted in a higher correlation coefficient (*r*^2^ > 0.98) than the Freundlich isotherm (*r*^2^ < 0.81). Secondly, the *q*_m_ values (87.39, 91.53, 96.06 and 99.56 mg g^−1^ at 25, 30, 35 and 40 °C, respectively) obtained using the Langmuir isotherm coincided well with the experimental values. Therefore, it can be said that the sorption process was mainly monolayer sorption of Pb^2+^ onto the homogenous surface of PMBY.

**Table 3:** Fitting results of adsorption isotherms based upon Langmuir and Freundlich models

*T* (°C)	*q*_e_ (mg g^−1^)	Langmuir			Freundlich		
		*q*_m_ (mg g^−1^)	*K*_L_ (L mg^−1^)	*r* ^2^	*K*_F_ [(mg g^−1^) (L mg^−1^)^1/n^]	*n*	*r* ^2^
25	84.26	87.39	0.2411	0.9857	38.6079	6.0295	0.7541
30	88.69	91.53	0.2834	0.9915	41.5128	6.1472	0.7627
35	93.85	96.06	0.3383	0.9959	44.5716	6.2263	0.7825
40	98.78	99.56	0.4922	0.9883	49.3750	6.6287	0.8004

Consequently, the Langmuir isotherm was further analyzed using the dimensionless constant, which was named as the equilibrium parameter or separation factor, and expressed as *R*_L_. *R*_L_ can be calculate using eqn (11).^6,8^11
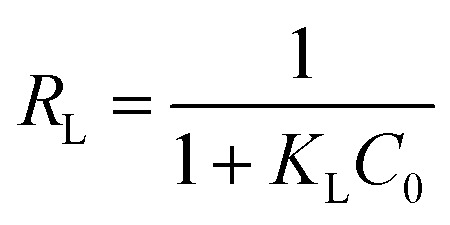


Various *R*_L_ values represent four kinds of adsorption characteristics, which are as follows: unfavorable (*R*_L_ > 1), linear (*R*_L_ = 1), favorable (0 < *R*_L_ < 1) and irreversible (*R*_L_ = 0)

**Fig. 6:**
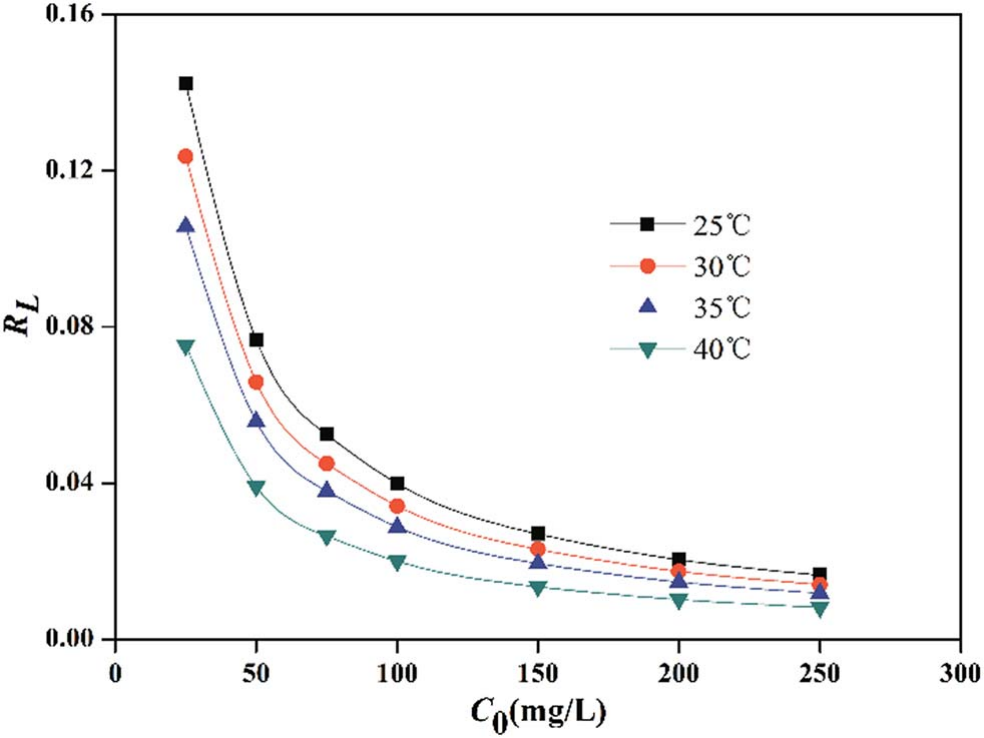
Langmuir separation factor (*t* = 15 min, *V* = 100 mL, PMBY dosage = 0.08 g, pH = 5.0 and *C*_0_ = 25–250 mg L^−1^).

Based upon the temperature and initial lead ion concentrations used in this work, *R*_L_ values were calculated, and it was found that, all of them ranged between 0–1 (Fig. 6), confirming that the sorption of Pb^2+^ by PMBY was favorable.

#### 3.2.5 Thermodynamic study

Various thermodynamic parameters, such as change in Gibbs free energy Δ*G* (kJ mol^−1^), change in enthalpy Δ*H* (kJ mol^−1^) and change in entropy Δ*S* (J (mol^−1^ K^−1^) were calculated using equilibrium data under different temperature conditions (25, 30, 35 and 40 °C). Δ*G*, Δ*H*, and Δ*S* are calculated using eqn (12), (13), and (14), respectively.

12 Δ*G* = −*NT* ln *K*

13
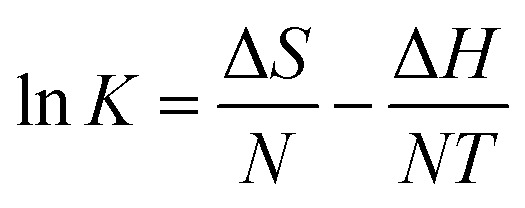

**Fig. 7:**
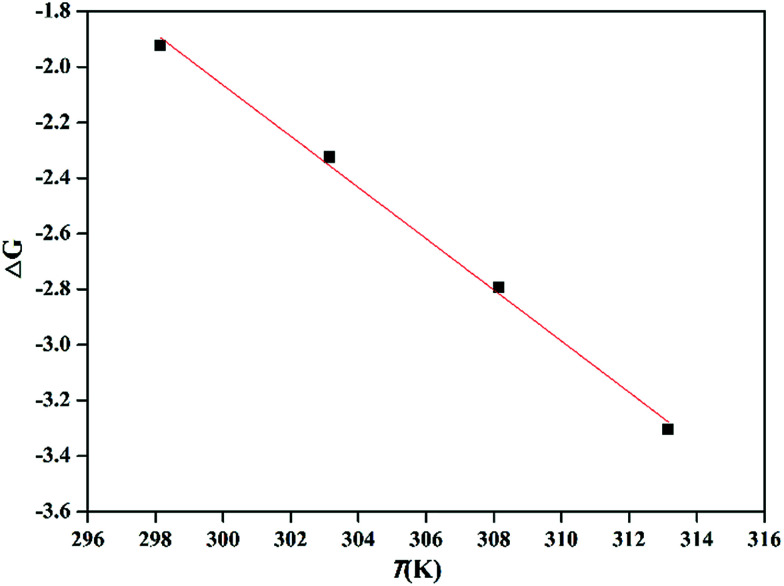
Plot of Δ*G* and *T* for the adsorption of Pb^2+^ using PMBY (*C*_0_ = 100 mg L^−1^, pH = 5.0, PMBY dosage = 0.08 g, *V* = 100 mL, *t* = 15 min and *T* = 25, 30, 35 and 40 °C).

14 Δ*G* = Δ*H* − *T*Δ*S*

where *N* is the universal gas constant 8.314 J (mol^−1^ K^−1^) and *T* is the temperature (K). In addition, *K* is the equilibrium constant at temperature *T*. Δ*S* and Δ*H* values can be obtained from the slope and intercept (respectively) of the graph drawn between Δ*G* and *T* values, and which is shown in Fig. 7. The values of the thermodynamic parameters are presented in Table 4.

**Table 4:** Thermodynamic parameters for the adsorption of Pb^2+^ by PMBY

*T* (°C)	Δ*G* (kJ mol^−1^)	Δ*H* (kJ mol^−1^)	Δ*S* (J (mol^−1^ K^−1^)
25	−1.92	25.56	92.09
30	−2.32		
35	−2.79		
40	−3.30		

Under different temperature conditions, the negative values of Δ*G* demonstrate that the adsorption of Pb^2+^ using PMBY was spontaneous, while the decreasing values of Δ*G* with increasing temperature (from 25 to 40 °C) reveal that the elevated temperature can promote the binding of Pb^2+^ onto the surface of PMBY sorbent. The positive values of Δ*H* confirm that the adsorption process was endothermic, and the sorption involved chemisorption as higher temperatures can promote the dissolution of lead ions and reduce the protonation of surface functional groups of the adsorbent to facilitate the chelation between Pb^2+^ and PMBY.^8^ The positive value of Δ*S* show that the randomness increased during the reaction, which was due to the destruction of hydration shell formed by water molecules on the surface of PMBY as the Pb^2+^ was bound on PMBY to make a number of water molecules enter the solution. All the thermodynamic parameters reflect that the bio-sorbent PMBY has an excellent affinity for Pb^2+^.

### 3.3 Adsorption mechanism

**Fig. 8:**
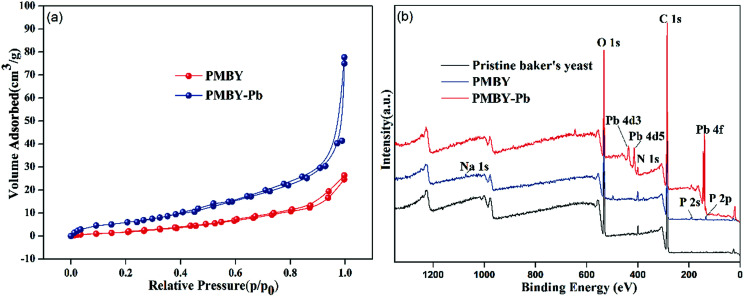
(a) N_2_ adsorption–desorption isotherms of PMBY and PMBY-Pb, (b) XPS analysis of pristine baker's yeast, PMBY and PMBY-Pb.

Nitrogen adsorption–desorption isotherms were constructed at −196.15 °C and were applied to calculate the specific surface area using the multipoint BET method. The nitrogen isotherms of the adsorbent PMYB before and after the adsorption (PMBY-Pb) are shown in Fig. 8a. The isotherm of PMYB and PMBY-Pb could be described as a Type IV isotherm, indicating that the PMBY and PMBY-Pb are mesoporous materials. The BET surface areas of PMBY and PMBY-Pb were calculated to be 6.140 and 40.686 m^2^ g^−1^. The BJH average pore size distribution of PMBY and PMBY-Pb were estimated using the desorption data, and the pore size was found to be 7.586 and 11.216 nm, respectively. After the adsorption, the surface area and pore size of PMBY-Pb were substantially increased compared to those of PMBY before the adsorption, thus indicating that PMBY had a great swelling power when it was dissolved in water. This swelling power could be attributed to the presence of phosphate groups in the PMBY, which possessed more water holding capacity and led to higher adsorption performance of PMYB for Pb^2+^. This result is in accordance with the findings reported by Qintie Lin *et al.* and Lin Qin-lu.^29,49^

After the adsorption of lead ions, there were large number of bright precipitates on the surface of PMBY, while the composites displayed a dense and compact structure (Fig. 2c and e). The EDS pattern (Fig. 2d and f) showed that a new peak of Pb appeared, while that of Na disappeared on PMBY-Pb compared to the PMBY. These changes illustrated that the lead ions were indeed adsorbed on the surface of PMBY through the mechanism of ion-exchange. Furthermore, comparing the FTIR spectra of PMBY and PMBY-Pb (shown in Fig. 3a), two new peaks at 1010.17 and 657.69 cm^−1^ were assigned to P–O–Pb and metal–oxygen (metal-hydroxide), respectively.^27,50^ The characteristic peaks of phosphate group obviously shifted or became weaker, which demonstrated that the removal of Pb^2+^ was mainly due to the phosphate groups. The adsorption mechanism was further investigated using XPS analysis.

**Fig. 9:**
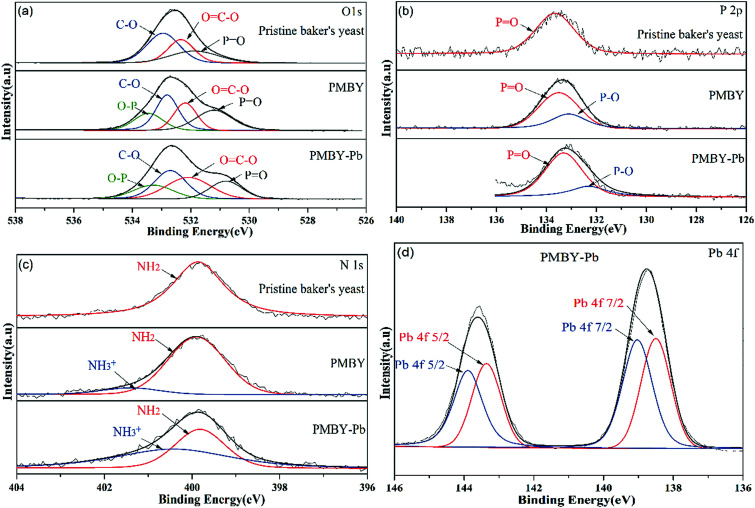
High-resolution spectra of O1s (a), P2p (b) and N1s (c) for the pristine baker's yeast, PMBY and PMBY-Pb, and the Pb 4f XPS spectra of PMBY-Pb (d).

The XPS spectra of pristine baker's yeast, PMBY and PMBY-Pb are displayed in Fig. 8b. Both the phosphorus and lead were observed obviously (Fig. 8b), indicating that the phosphorylation reaction had occurred, and that the lead ions were adsorbed to the surface of PMBY. The high-resolution spectra of O1s, P2p, N1s and Pb 4f are shown in Fig. 9, whereas the proposed components and their binding energies are presented in Table 5. Comparing the O1s, P2p and N1s spectra of pristine baker's yeast and PMBY (Fig. 9a, b and c), some novel peaks emerged beside the original peaks of O-, P- and N-containing functional groups in pristine baker's yeast. The new peaks confirmed that phosphate groups were introduced on the surface of pristine baker's yeast. The different binding energies of C–O, O=C–O, –NH_2_ from PMBY and pristine baker's yeast illustrated that the hydroxyl, carboxyl and amino groups reacted with the phosphate. The results were found to be consistent with the FTIR characterization.

**Table 5:** Proposed components and their binding energies for pristine baker's yeast, PMBY and PMBY-Pb

Valence state	Pristine baker's yeast		PMYC		PMYC-Pb	
	Proposed component	Binding energy (eV)	Proposed component	Binding energy (eV)	Proposed component	Binding energy (eV)
O1s	C–O^19,24^	532.95	C–O	532.81	C–O	532.70
	O=C–O^19,24^	532.34	O=C–O	532.19	O=C–O	532.08
	P=O^27^	531.88	P=O	531.21	P=O	530.80
	—	—	O–P^28^	533.45	O–P	533.28
P2p	P=O^28^	133.56	P=O	133.50	P=O	133.31
	—	—	P–O^27,28^	133.11	P–O	132.3
N1s	NH_2_ (ref. 34)	399.86	NH_2_	399.9	NH_2_	399.82
	—	—	NH^3+^ (ref. 51)	401.4	NH^3+^	400.42
Pb 4f	—	—	—	—	Pb 4f 5/2 (ref. 27)	143.19, 142.66
	—	—	—	—	Pb 4f 7/2 (ref. 27)	138.33, 137.8

After the adsorption, the peaks of O-, P- and N-containing functional groups in PMBY showed variations in terms of binding energy. However, the reduction binding energies of P=O and P–O were the most obvious, revealing that the phosphate groups were mainly involved in the adsorption of lead.

The Pb 4f spectrum for PMBY-Pb is depicted in Fig. 9d. The peaks at around 140 eV were assigned to Pb 4f due to the adsorption of Pb^2+^. The peaks at 143.19 and 138.33 could be assigned to Pb^2+^, indicating that the lead was loaded on the surface of PMBY through chelation. Moreover, the Pb 4f peaks were centered at 142.66 eV and 137.8 eV, which suggested that Pb^2+^ may have been absorbed in PMBY in the form of Pb–O–P through ion-exchange process. According to the XPS spectra of PMBY and PMBY-Pb, the Na peak disappeared in the spectra of PMBY-Pb, indicating that the adsorption process of PMBY for Pb^2+^ followed ion-exchange. This result was also confirmed by the results of SEM-EDS. In addition, it is well-known that the metal cations are typical Lewis acids and that the phosphate groups with low acid–base ionization equilibrium constant (p*K*_a_ = 1–2) show typical Lewis base properties in a wide range of pH values.^27^ Therefore, based upon the Lewis acid–base theory, lead ions can interact with the phosphate groups through chelation and electrostatic interaction. Due to the successful introduction of phosphate groups and the interaction (ion-exchange, chelation and electrostatic attraction) between the phosphate groups and Pb^2+^, the adsorption performance of PMBY for Pb^2+^ significantly improved.

**Fig. 10:**
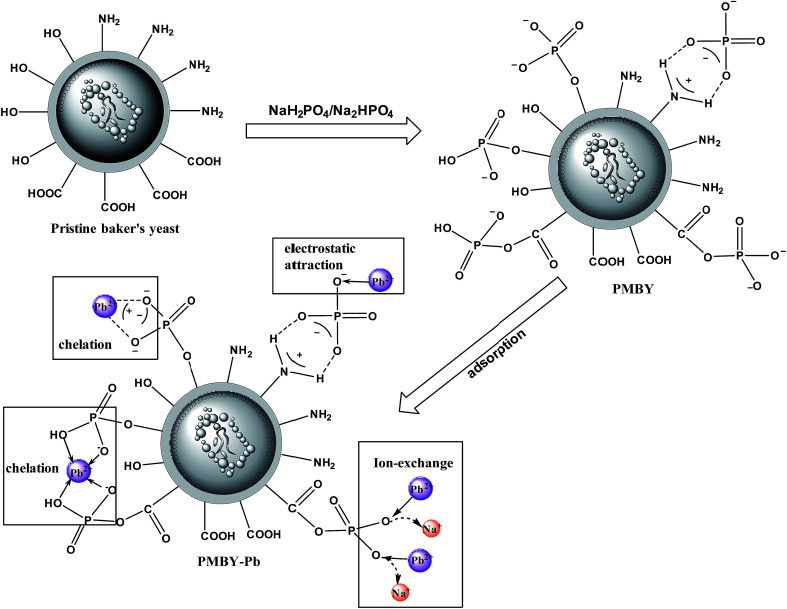
Reaction scheme and schematic of adsorption mechanism of Pb^2+^ by PMBY.

Fig. 10 shows the reaction scheme and the proposed schematic of the adsorption mechanism of PMBY for Pb^2+^. Firstly, the surface functional groups of baker's yeast cell walls, such as hydroxyl, carboxyl and amine groups, reacted with NaH_2_PO_4_/Na_2_HPO_4_. The detailed synthesis is shown in Fig. 1. The phosphate groups were linked to the yeast through substitution reaction or the ligand exchange process between the O–H group of hydroxyl groups and carboxylic acids, and the phosphate. Additionally, the amine groups and phosphate groups could react through electrostatic attraction and hydrogen bonding. After this reaction, the novel PMBY bio-sorbent was obtained and used to remove Pb^2+^ from aqueous solution. The phosphate groups, which were grated into the surface of pristine baker's yeast played a significant role during the adsorption process. As shown in Fig. 10, the PMBY efficiently removed Pb^2+^ from aqueous solution. The process mainly depended upon these interactions (ion-exchange, chelation and electrostatic attraction) between the phosphate groups and Pb^2+^. The adsorption mechanism could be confirmed using SEM, FTIR and XPS analyses.

### 3.4 Regeneration of PMBY

**Fig. 11:**
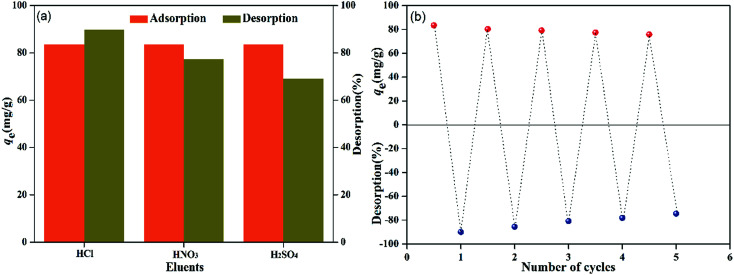
Regeneration of PMBY.

A good adsorbent should not only possess high adsorption affinity, but also show excellent regeneration property. These characteristics are of great importance for decreasing its production and application costs. The adsorption–desorption study was done using different acid solvents (0.01 M HCl, HNO_3_ and H_2_SO_4_).^8^ For the process, 0.08 g of PMBY was added to 100 mL of 100 mg L^−1^ Pb^2+^ solution in conical flasks, and the pH was adjusted to 5.0. Then, the mixture was shaken using a rotary shaker (speed of 150 rpm) for 15 min at 30 °C. Subsequently, the Pb-loaded PMBY (PMBY-Pb) was treated using 100 mL of the abovementioned acid solvents under the aforementioned conditions for 120 min. Then, the mixtures were filtered, and the filtrate was used to determine the Pb^2+^ concentration using AAS. The results are shown in Fig. 11.

The order of desorption for Pb^2+^ was found to be: HCl (89.85%) > HNO_3_ (77.42%) > H_2_SO_4_ (69.06%) (Fig. 11a). The better recovery of Pb^2+^ in 0.01 M HCl was due to the smaller sized Cl^−^ ions in comparison to the NO^3−^ and SO_4_^2−^ ions.^8^ Hence, the recyclability of PMBY for the adsorption of Pb^2+^ was confirmed using 0.01 M HCl solution. As can be seen from Fig. 11b, after five regeneration cycles, PMBY still exhibited 90.77% of the original adsorption capacity. Therefore, it can safely be said that the adsorption efficiency of PMBY towards Pb^2+^ was still satisfactory after several regeneration cycles, whereas HCl was used as the eluent during these regeneration experiments. All these results suggested that PMBY could act as a renewable and efficient adsorbent for the remediation of wastewater containing Pb^2+^.

## 4. Conclusions

In this work, phosphate-modified baker's yeast (PMBY) was successfully synthesized using phosphate treatment of baker's yeast combined with the dry-heating. The surface morphology of PMBY exhibited irregular shape and a large volume of pores, which were beneficial for the adsorption of Pb^2+^. The results of FTIR, elemental analysis and XPS showed that phosphate groups were indeed introduced onto the yeast, whereas the hydroxyl, carboxyl and amine groups of pristine baker's yeast participated in the phosphorylation process. The efficient adsorption of Pb^2+^ by PMBY mainly depended on the additional phosphate groups, which fixed the Pb^2+^ ions through ion-exchange, electrostatic attraction and chelation. It was found that the adsorption capacity of PMBY was superior to that of the pristine baker's yeast, while the adsorption process was very rapid and could attain equilibrium in around 3 min. The results from adsorption kinetic and isotherm analyses revealed that the Pb^2+^ adsorption process could be well described by pseudo-second-order kinetics and Langmuir isotherm model, respectively. Furthermore, the adsorption process of Pb^2+^ on the surface of PMBY was spontaneous and endothermic. The main Pb^2+^ adsorption mechanism of PMBY was based upon ion-exchange, electrostatic interaction and chelation between the phosphate groups and Pb^2+^. In addition, the bio-sorbent PMBY showed excellent regeneration performance. 0.01 M HCl was used as the eluent in regeneration experiments. Finally, the results of the study show that PMBY has significant potential to be used as an efficient and useful adsorbent for the removal of heavy metal ions from industrial wastewater.

## Conflicts of interest

There are no conflicts to declare.

## Supplementary Material
